# Short apneas and periodic breathing in preterm infants in the neonatal intensive care unit—Effects of sleep position, sleep state, and age

**DOI:** 10.1111/jsr.14253

**Published:** 2024-06-04

**Authors:** Georgina Plunkett, Stephanie Yiallourou, Aimee Voigt, Aishah Segumohamed, Kelsee Shepherd, Rosemary Horne, Flora Wong

**Affiliations:** ^1^ The Ritchie Centre Hudson Institute of Medical Research Melbourne Victoria Australia; ^2^ The Department of Paediatrics Monash University Melbourne Victoria Australia; ^3^ The Turner Institute for Brain and Mental Health, School of Psychological Sciences, Monash University Melbourne Victoria Australia; ^4^ Monash Newborn, Monash Children's Hospital Melbourne Victoria Australia

**Keywords:** NICU, periodic breathing, preterm infants, prone sleep position, short apneas

## Abstract

This observational study investigated the effects of sleep position and sleep state on short apneas and periodic breathing in hospitalized preterm infants longitudinally, in relation to postmenstrual age. Preterm infants (25–31 weeks gestation, *n* = 29) were studied fortnightly after birth until discharge, in prone and supine positions, and in quiet sleep and active sleep. The percentage of time spent in each sleep state (percentage of time in quiet sleep and percentage of time in active sleep), percentage of total sleep time spent in short apneas and periodic breathing, respectively, the percentage of falls from baseline in heart rate, arterial oxygen saturation and cerebral tissue oxygenation index during short apneas and periodic breathing, and the associated percentage of total sleep time with systemic (arterial oxygen saturation < 90%) and cerebral hypoxia (cerebral tissue oxygenation index < 55%) were analysed using a linear mixed model. Results showed that the prone position decreased (improved) the percentage of falls from baseline in arterial oxygen saturation during both short apneas and periodic breathing, decreased the proportion of infants with periodic breathing and the periodic breathing‐associated percentage of total sleep time with cerebral hypoxia. The percentage of time in quiet sleep was higher in the prone position. Quiet sleep decreased the percentage of total sleep time spent in short apneas, the short apneas‐associated percentage of falls from baseline in heart rate, arterial oxygen saturation, and proportion of infants with systemic hypoxia. Quiet sleep also decreased the proportion of infants with periodic breathing and percentage of total sleep time with cerebral hypoxia. The effects of sleep position and sleep state were not related to postmenstrual age. In summary, when sleep state is controlled for, the prone sleeping position has some benefits during both short apneas and periodic breathing. Quiet sleep improves cardiorespiratory stability and is increased in the prone position at the expense of active sleep, which is critical for brain maturation. This evidence should be considered in positioning preterm infants.

## 1 INTRODUCTION

Preterm birth is associated with immature respiratory control, which can manifest as short apneas (sAPN), which are pauses in breathing for 3–10s (Berry et al., 2012), and periodic breathing (PB), which is defined as ≥ 3 sequential apneas lasting ≥ 3 s each being separated by ≤ 20 s of normal respiration (Berry et al., 2012). Traditionally, sAPN and PB were considered benign developmental conditions amongst preterm infants as the apneas are short (Zhao et al., 2011). However, sAPN and PB often persist into infancy, and can be associated with significant falls in blood pressure, heart rate (HR), arterial oxygen saturations (SpO_2_) and cerebral tissue oxygenation index (TOI) in both preterm and term born infants after hospital discharge (Albani et al., 1985; Horne et al., 2018). Repetitive hypoxia/reoxygenation following sAPN and PB may initiate a proinflammatory cascade predisposing to cerebral injury and adverse neurodevelopment (Martin et al., 2011; Yee, Siriwardhana, Nixson, et al., 2023).

The sAPN and PB are reported to occur in 60%–100% of preterm infants before term equivalent age, and PB often causes prolonged desaturations (Oliveira et al., 2004; Patel et al., 2016). Prone positioning has been shown to reduce oxygen desaturations (Kurlak et al., 1994; Rivas‐Fernandez et al., 2016), bradycardias (Kurlak et al., 1994), apnea density (Kurlak et al., 1994) and ventilatory requirements in preterm infants (Hadaya & Benharash, 2020; Rivas‐Fernandez et al., 2016). As a result, preterm infants are often slept prone in neonatal units (Shepherd et al., 2021), and the practice may continue after hospital discharge (Vernacchio et al., 2003), contrary to the clinical recommendation that clinically stable preterm infants from 32 weeks postmenstrual age (PMA) are positioned supine for sleep (Moon, 2016). Notably, previous studies have shown that when sleep state was controlled for, both SpO_2_ and frequency of apneas were not affected by the prone position in preterm infants (Elder et al., 2005; Elder et al., 2011). We have previously reported that in very preterm infants, the quiet sleep (QS) state had a more marked effect than the prone position in reducing the moderate to severe cardiorespiratory events (Shepherd et al., 2019). Active sleep (AS) and QS are the two main sleep states in preterm infants, with AS being associated with higher cerebral oxygen consumption (Fyfe et al., 2014; Shepherd et al., 2019) and greater cardiorespiratory variability (Gaultier & Gallego, 2005), including increased PB (Elder et al., 2011). Despite the high incidence of sAPN and PB in preterm infants prior to hospital discharge, their physiological effects in relation to sleep position, sleep state and PMA remain unknown.

In this study, we aimed to determine the effects of sleeping position (prone versus supine), sleep state (AS versus QS) and PMA on the frequency and physiological consequences (HR, SpO_2_ and TOI) of sAPN and PB, using a longitudinal design. We hypothesized that prone sleeping, QS and increasing PMA would be associated with improved respiratory stability as compared with the supine position and AS.

## 2 METHODS

Ethical approval was obtained from the Monash Health and Monash University human research ethics committees (Ref no: 12217B), and written informed parental consent was obtained.

### 2.1 Subjects

Preterm infants born between 25 and 31 weeks of gestational age (GA) were studied at Monash Newborn. Exclusion criteria included intrauterine growth restriction, indwelling umbilical catheters, major congenital abnormalities or major brain pathologies. Clinical management was up to the discretion of the attending physician. The changes in cerebral oxygenation and cardiorespiratory parameters in relation to sleep positions and prolonged apneas (>10 s) in this cohort have been published previously (Shepherd et al., 2019; Shepherd et al., 2020; Shepherd et al., 2021).

### 2.2 Study protocol

Infants were studied longitudinally on a fortnightly basis following parental consent from day 7 or when considered medically stable and continued until term‐equivalent age or discharge. The preterm infants were studied using daytime polysomnography for 2–4 hr in duration with time distributed between prone and supine positions at each study, as described previously (Shepherd et al., 2019). During each study, to avoid interference with the regular nursing care, infants were studied in both positions in the prone/supine or supine/prone sequence according to the nursing care schedule. In the prone position, the head was rotated to one side, and in the supine position, the head was retained in the midline. During the study, additional care procedures were minimized, while feeds were given as per the infant's schedule via nasogastric tube. HR and respiratory effort and rate were recorded using electrocardiogram electrodes (Covidien, USA) and a respiratory impedance belt (Respiband, ADInstruments, Australia), respectively. An oximeter probe was placed on the right wrist to measure pre‐ductal SpO_2_ (2 s averaging time; Masimo, USA). Adjustments in respiratory support, ventilation and fraction of inspired oxygen (FiO_2_) were made according to clinical protocol to maintain SpO_2_ between 90% and 95%. During the study, care procedures were minimized. Cerebral tissue oxygenation, expressed as TOI (%), was measured continuously by near infrared spectroscopy (NIRO 200NX, Hamamatsu Photonics KK, Japan), with two optodes placed over the temporoparietal region (Shepherd et al., 2019). TOI represents the ratio between oxy‐ and total haemoglobin in the measured brain region. HR, SpO_2_, respiratory rate and TOI were simultaneously recorded (Powerlab, ADInstruments, Australia) at a sampling rate of 400 Hz. Sleep state was continuously scored at the bedside in 30‐s epochs using validated behavioural criteria, including HR, breathing patterns and presence or absence of eye movements (Lilia Curzi‐Dascalova, 1996; Shepherd et al., 2019). QS was characterized by regular respiratory and HR patterns and the absence of body and eye movements, whereas AS was characterized by eye and body movements, irregular HR and respiratory patterns. Periods where features of both AS and QS were exhibited were marked as indeterminate sleep, and these epochs were added to AS for data analysis.

### 2.3 Data analysis

Data were analysed using LabChart software (ADInstruments, Australia). All study recordings were visually examined for episodes of sAPN and PB. The duration of PB was measured from the beginning of the first apnea until the end of the last apnea. For each respiratory event, an artefact‐free continuous baseline of 10 s was marked within a 20‐s period before the beginning of an apnea. A “post‐event” baseline of 15 s was taken following the respiratory event to account for any lag time in physiological changes (Decima et al., 2015). We only included respiratory events where the baseline, event and post‐baseline were artefact‐free (no movement artefact or disruption from sleep). The percentage time spent in each sleep state (QS% and AS%), percentages of total sleep time (%TST) spent with sAPN and PB, respectively, and the duration of sAPN and PB events were calculated. Due to the cyclical changes in HR, SpO_2_ and TOI that occur during repetitive apneas, percentage changes from baseline averaged over each respiratory event would not accurately reflect the extent of the falls in these parameters. Therefore, the nadir in HR, SpO_2_ and TOI for each respiratory event was used to calculate the maximum change from the baseline of HR (∆HR%), SpO_2_ (∆SpO_2_%) and TOI (∆TOI%). Percentages of time spent with SpO_2_ < 90% and TOI < 55% during respiratory events were calculated at each study. We used these cut‐offs as the SpO_2_ target in our neonatal unit is >90% (Schmidt & Whyte, 2020), and an SpO_2_ of 85%–89% has been associated with increased mortality and morbidity (Tarnow‐Mordi et al., 2016). Cerebral oxygenation of < 55% has been associated with adverse neurodevelopmental outcomes in preterm infants (Alderliesten et al., 2019). All data were averaged for each sleep state and position in each infant, at each PMA group and chronological age (postnatal week).

### 2.4 Statistical analysis

Cardiorespiratory parameters listed above were analysed using a linear mixed model approach (SPSS Version 29). This statistical approach increases the robustness of our findings, in light of the characteristics of the dataset including repeated measures, missing at random data (e.g. no occurrence of respiratory events in a particular position or sleep state, or infants being discharged at different ages). Three models were used with the following fixed effects: (1) position, sleep state, PMA and position × state interaction; (2) position, sleep state, PMA and position × PMA interaction; and (3) position, sleep state, chronological age and position × chronological age interaction. Participant ID was used as a random effect, and PMA or chronological age was used as repeated measures in all models. The linear mixed model was also used to analyse the QS%, AS% and FiO_2_ for each position with fixed effects as: (1) position and PMA; and (2) position and chronological age. Data are presented as mean ± SEM. A chi‐squared test was used to analyse proportions of infants that exhibited events (either sAPN or PB), and the proportion of infants who spent time in systemic and cerebral hypoxia (SpO_2_ < 90% and TOI < 55%, respectively) due to sAPN or PB, in each sleep state–position combination at each PMA. Significance was accepted at *p* < 0.05.

## 3 RESULTS

Twenty‐nine preterm infants (15F/14M), who each had at least three fortnightly studies spanning the first 6 postnatal weeks, were included with a total of 109 studies analysed. Infants had a median (range) GA at birth of 28 (25–31) weeks, with a median (range) birth weight of 1075 (715–1776) g. All infants received caffeine therapy as per clinical protocol. Other demographic characteristics including Apgar scores, cardiorespiratory diagnosis and treatments are presented in Table 1. The number of infants at each PMA, their ventilation requirements during the studies, and incidence of respiratory events in each sleep position and sleep state are presented in Table 2. The infants had relatively mild respiratory dysfunction as evidenced by their FiO_2_ requirement of < 30% over the study period (Table 2).

**TABLE 1:** Demographic characteristics of the infants studied (*n* = 29).

Female, *n* (%)	15 (52%)
Twin, *n* (%)	8 (27%)
Triplet, *n* (%)	1 (3%)
Received antenatal corticosteroids, *n* (%)	28 (96%)
Respiratory distress syndrome, *n* (%)	27 (93%)
Treated for presumed sepsis or proven late‐onset sepsis after 1 week, *n* (%)	29 (100%)
Haemodynamically significant patent ductus arteriosus requiring treatment, *n* (%)	2 (7%)
Intraventricular haemorrhage (Grade 1 or 2) on cranial ultrasound, *n* (%)	3 (10%)
Apgar 1 min, median (range)	6 (3–9)
Apgar 5 min, median (range)	8 (6–9)

**TABLE 2:** Ventilatory requirements and incidence of sAPN/PB at each PMA.

	Ventilatory requirements				Incidence of short apnea (sAPN) or periodic breathing (PB)				
Postmenstrual age (weeks)	IMV	CPAP	HF	SVIRA		AS‐S	QS‐S	AS‐P	QS‐P
25–26 (*N* = 3)	‐	3	‐	‐	sAPN	3 (100%)	3 (100%)	3 (100%)	3 (100%)
					PB	3 (100%)	2 (67%)	2 (67%)	1 (33%)
27–28 (*N* = 11)	2 (1)	8 (3)	‐	1	sAPN	11 (100%)	11 (100%)	11 (100%)	10 (91%)
					PB	10 (91%)	7 (64%)	10 (91%)	5 (45%)^#^
29–30 (*N* = 20)	‐	11 (4)	6 (2)	3	sAPN	20 (100%)	19 (95%)	19 (95%)	19 (95%)
					PB	19 (95%)	11 (55%)^##^	14 (70%)*	10 (50%)
31–32 (*N* = 29)	‐	11 (2)	4 (2)	14	sAPN	29 (100%)	26 (90%)	29 (100%)	27 (93%)
					PB	25 (86%)	19 (66%)	21 (72%)	10 (34%)*^##^
33–34 (*N* = 27)	‐	6	4	17	sAPN	27 (100%)	25 (93%)	27 (100%)	25 (93%)
					PB	24 (89%)	18 (67%)^#^	16 (59%)*	11 (41%)
35–36 (*N* = 19)	‐	‐	3	16 (^)	sAPN	19 (100%)	19 (100%)	19 (100%)	18 (95%)
					PB	14 (74%)	10 (53%)	12 (63%)	11 (58%)

*Note*: Values for ventilatory requirements represent number of infants on each type of respiratory support in air, plus (*n*) infants on FiO_2_ > 0.21. The incidence indicates the number of infants (% of total of number of infants studied) who had sAPN or PB in each sleep position and state during the study. * = position, *p < 0.05; ^#^ = sleep state, ^#^ *p* < 0.05, ^##^ *p* < 0.01; ^ = one infant on low flow oxygen.

Abbreviations: AS‐S, active sleep in supine position; AS‐P, active sleep in prone position; CPAP, continuous positive airway pressure; HF, high flow; IMV, intermittent mechanical ventilation; PB, periodic breathing; PMA, postmenstrual age; QS‐P, quiet sleep in prone position; QS‐S, quiet sleep in supine position; sAPN, short apneas; SVIRA, self‐ventilating in room air.

**FIGURE 1:**
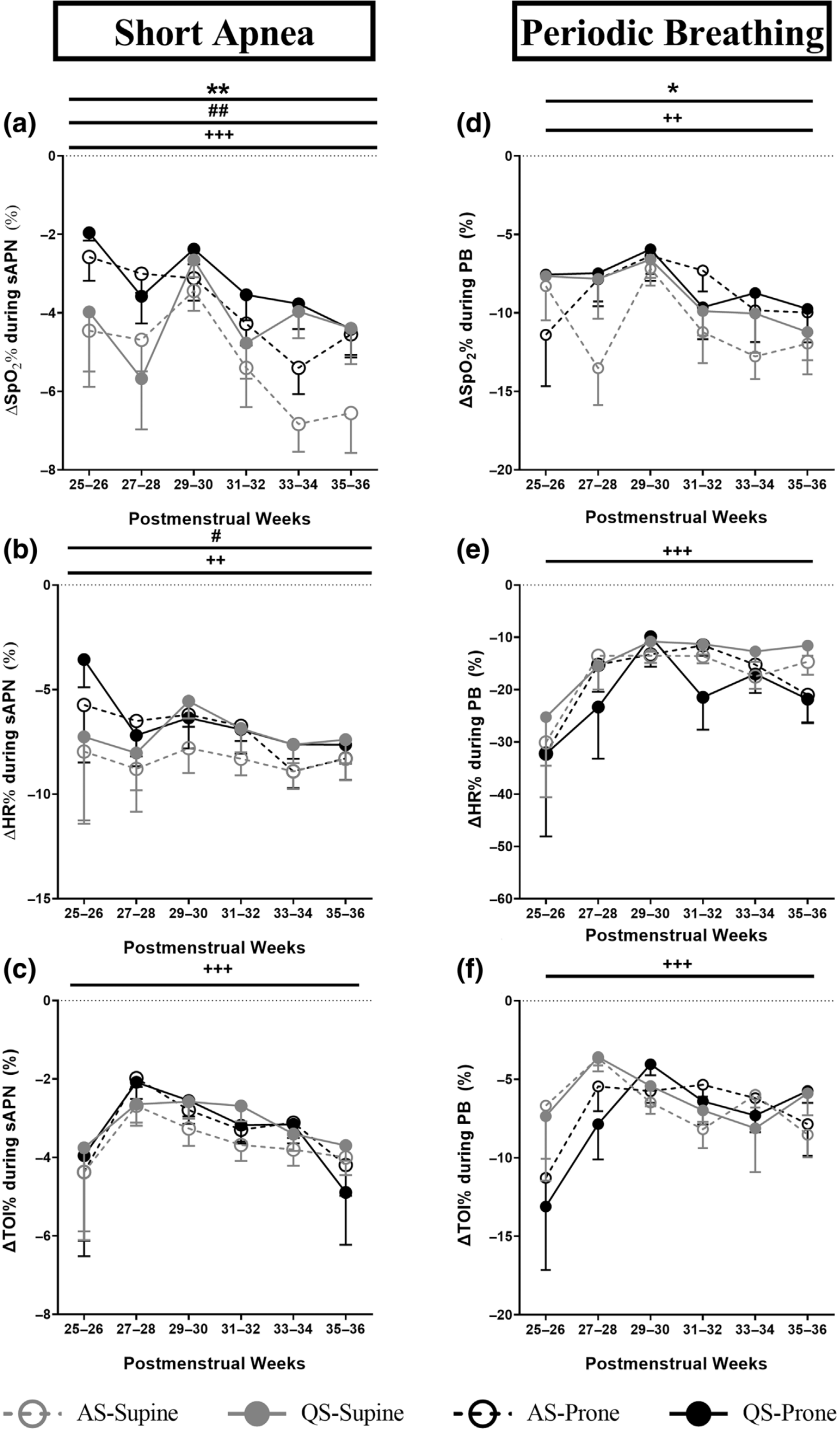
Effect of sleep position, sleep state and PMA on percentage change from baseline in SpO_2_ (ΔSpO_2_%), HR (ΔHR%) and cerebral TOI (ΔTOI%) in sAPN (a, b and c, respectively) and PB (d, e and f, respectively). Values are mean ± SEM. *p < 0.05 overall effect of sleep position; **p < 0.01 overall effect of sleep position; ^#^ *p* < 0.05 overall effect of sleep state; ^##^ *p* < 0.01 overall effect of sleep state; ^++^ *p* < 0.01 overall effect of PMA; ^+++^ *p* < 0.001 overall effect of PMA. HR, heart rate; PB, periodic breathing; PMA, postmenstrual age; sAPN, short apneas; SpO_2_, arterial oxygen saturation; TOI, tissue oxygenation index.

**FIGURE 2:**
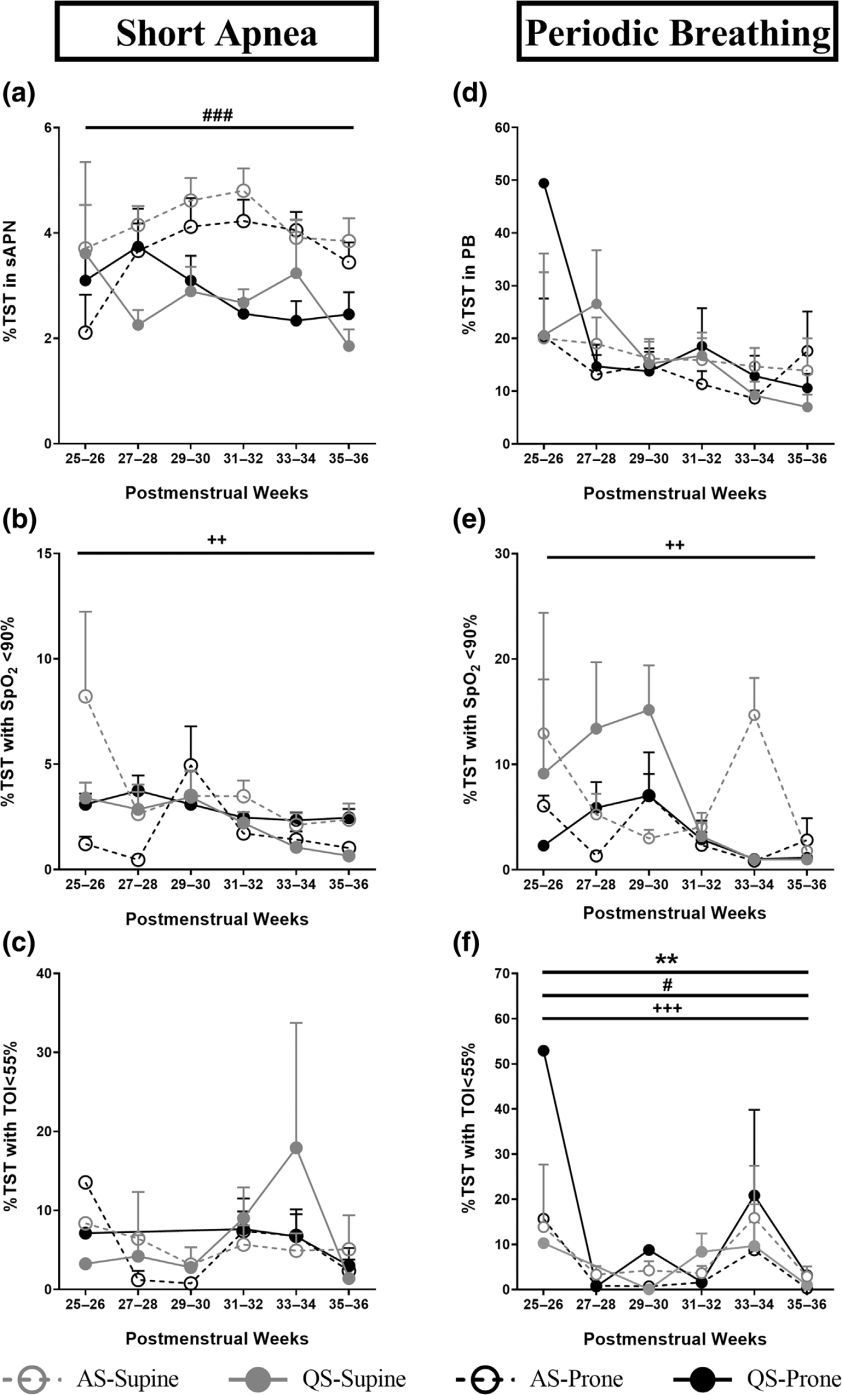
Effect of sleep position, sleep state and PMA on %TST spent in sAPN and PB (a, d), and the respective %TST in systemic (b, e) and cerebral hypoxia (c, f). Values are mean ± SEM. **p < 0.01 overall effect of sleep position; ^#^ *p* < 0.05 overall effect of sleep state; ^###^ *p* < 0.001 ^++^ *p* < 0.01 overall effect of PMA; ^+++^ *p* < 0.001 overall effect of PMA. PB, periodic breathing; PMA, postmenstrual age; sAPN, short apneas; %TST, percentage of total sleep time.

A total of 5160 sAPN and 1250 PB episodes were analysed. The baseline physiological parameters and durations of sAPN/PB are presented in Table 3. Mixed model analyses of the maximum ∆HR%, ∆TOI% and ∆SpO_2_%, as well as %TST spent in sAPN/PB, %TST spent in SpO_2_ < 90% or TOI < 55% are summarized in Table 4 (statistical results), and illustrated in Figures 1 and 2 (actual values of the physiological data) for sAPN and PB, respectively.

**TABLE 3:** Baseline values of the physiological parameters before the sAPN and PB episodes, and the TST in each position and episode durations for each PMA.

Postmenstrual age (weeks)			AS‐S	QS‐S	AS‐P	QS‐P
25–26 (*N* = 3)	TST (min)		55.84 ± 6.72		43.15 ± 5.94	
	Episode duration (s)	sAPN	4.89 ± 0.71	6.14 ± 0.06	5.40 ± 0.24	5.78 ± 1.00
		PB	61.70 ± 20.83	76.31, 45.76	66.56, 61.41	94.11
	Baseline HR (bpm)	sAPN	160.54 ± 8.67	157.54 ± 8.80	166.42 ±10.1	165.12 ± 11.4
		PB	155.32 ± 10.5	172.95, 138.3	180.18, 146.1	179.7
	Baseline SpO_2_ (%)	sAPN	90.02 ± 3.54	89.99 ± 3.38	95.53 ± 1.05	95.31 ± 1.24
		PB	91.63 ± 2.16	90.77, 97.33	96.08, 96.16	96.4
	Baseline TOI (%)	sAPN	62.52 ± 3.22	64.10 ± 3.14	64.81 ± 5.96	64.08 ± 3.74
		PB	63.19 ± 3.21	57.49, 68.46	55.20, 66.49	54.98
27–28 (*N* = 11)	TST (min)		69.69 ± 4.73		62.09 ± 5.96	
	Episode duration (s)	sAPN	5.65 ± 0.30	6.00 ± 0.46	5.49 ± 0.21	5.15 ± 0.26
		PB	61.26 ± 8.66	85.95 ± 13.45	50.23 ± 3.45	60.17 ± 4.06
	Baseline HR (bpm)	sAPN	156.05 ± 2.03	155.91 ± 2.27	157.61 ± 1.35	156.21 ± 1.65
		PB	155.93 ± 1.69	156.04 ± 2.45	159.21 ± 1.85	152.14 ± 2.23
	Baseline SpO_2_ (%)	sAPN	95.77 ± 0.83	94.09 ± 1.09	96.85 ± 0.50	96.94 ± 0.71
		PB	93.27 ± 2.63	96.55 ± 0.86	95.96 ± 0.46	95.77 ± 1.18
	Baseline TOI (%)	sAPN	65.25 ± 1.92	64.40 ± 1.81	65.35 ± 1.50	66.17 ± 1.28
		PB	63.05 ± 2.39	66.25 ± 1.92	64.47 ± 1.66	66.59 ± 2.13
29–30 (*N* = 20)	TST (mins)		64.99 ± 1.96		62.66 ± 2.70	
	Episode duration (s)	sAPN	5.08 ± 0.12	5.32 ± 0.18	5.16 ± 0.16	5.00 ± 0.18
		PB	56.56 ± 6.61	61.11 ± 8.11	55.46 ± 4.79	88.43 ± 16.81
	Baseline HR (bpm)	sAPN	161.56 ± 2.41	159.02 ± 2.72	160.68 ± 2.20	162.23 ± 2.41
		PB	161.79 ± 2.54	162.08 ± 2.80	159.08 ± 2.00	156.77 ± 3.94
	Baseline SpO_2_ (%)	sAPN	95.87 ± 0.66	96.34 ± 0.62	96.42 ± 0.75	96.66 ± 0.69
		PB	94.70 ± 0.99	95.00 ± 1.03	95.36 ± 1.11	93.23 ± 2.25
	Baseline TOI (%)	sAPN	67.49 ± 1.33	67.52 ± 1.41	67.51 ± 1.18	67.95 ± 1.15
		PB	67.19 ± 1.29	67.93 ± 2.05	67.21 ± 1.43	68.24 ± 2.79
31–32 (*N* = 29)	TST (min)		64.83 ± 1.73		64.07 ± 2.40	
	Episode duration (s)	sAPN	5.15 ± 0.11	5.16 ± 0.22	5.14 ± 0.14	4.98 ± 0.18
		PB	52.43 ± 5.80	71.66 ± 9.89	48 ± 3.80	63.91 ± 7.12
	Baseline HR (bpm)	sAPN	163.19 ± 2.07	160.40 ± 2.17	163.95 ± 1.60	162.39 ± 2.10
		PB	162.41 ± 2.22	161.15 ± 2.54	162.40 ± 2.05	160.34 ± 2.27
	Baseline SpO_2_ (%)	sAPN	95.39 ± 0.55	96.28 ± 0.57	96.25 ± 0.58	96.80 ± 0.60
		PB	95.79 ± 0.58	96.61 ± 0.67	96.36 ± 0.72	96.92 ± 1.08
	Baseline TOI (%)	sAPN	64.92 ± 1.20	65.18 ± 1.7	66.23 ± 1.41	67.02 ± 1.35
		PB	65.42 ± 1.32	64.93 ± 1.81	66.69 ± 1.69	69.80 ± 1.29
33–34 (*N* = 27)	TST (min)		65.57 ± 2.33		64.48 2.22	
	Episode duration (s)	sAPN	4.66 ± 0.08	4.88 ± 0.14	4.88 ± 0.10	5.20 ± 0.17
		PB	61.27 ± 6.93	50.37 ± 5.55	41.78 ± 1.90	52.46 ± 4.99
	Baseline HR (bpm)	sAPN	162.38 ± 1.42	157.88 ± 2.03	162.38 ± 1.44	159.59 ± 1.56
		PB	162.89 ± 1.84	156.88 ± 2.09	162.00 ± 2.36	158.20 ± 2.92
	Baseline SpO_2_ (%)	sAPN	96.81 ± 0.45	97.34 ± 0.38	97.41 ± 0.29	97.51 ± 0.49
		PB	96.27 ± 0.69	97.06 ± 0.68	95.20 ± 1.24	97.19 ± 0.75
	Baseline TOI (%)	sAPN	65.44 ± 1.20	66.52 ± 1.19	66.32 ± 1.19	67.20 ± 1.27
		PB	65.64 ± 1.15	65.56 ± 1.56	66.35 ± 1.83	66.35 ± 2.39
35–36 (*N* = 19)	TST (min)		68.94 ± 2.92		57.30 ± 1.87	
	Episode duration (s)	sAPN	4.49 ± 0.14	4.62 ± 0.16	4.47 ± 0.13	5.15 ± 0.20
		PB	44.77 ± 10.15	46.96 ± 7.91	61.15 ± 15.22	59.10 ± 8.61
	Baseline HR (bpm)	sAPN	162.75 ± 2.12	158.59 ± 1.44	162.52 ± 2.44	160.77 ± 2.40
		PB	161.79 ± 2.78	159.60 ± 2.62	158.07 ± 3.77	160.18 ± 3.24
	Baseline SpO_2_ (%)	sAPN	97.31 ± 0.59	97.88 ± 0.62	97.84 ± 0.39	98.29 ± 0.33
		PB	96.68 ± 0.93	96.91 ± 1.46	98.70 ± 0.30	98.36 ± 0.41
	Baseline TOI (%)	sAPN	65.13 ± 1.21	66..22 ± 1.21	67.88 ± 1.41	68.87 ± 1.47
		PB	64.79 ± 1.56	65.95 ± 1.21	70.21 ± 1.27	68.75 ± 1.96

*Note*: Values are presented as mean ± SEM for *n* ≥ 3. Individual data are presented when *n* < 3.

Abbreviations: AS‐S, active sleep in supine position; AS‐P, active sleep in prone position; HR, heart rate; PB, periodic breathing; PMA, postmenstrual age; QS‐P, quiet sleep in prone position; QS‐S, quiet sleep in supine position; sAPN, short apneas; SpO_2_, arterial oxygen saturation; TOI, tissue oxygenation index; TST, total sleep time.

**TABLE 4:** Mixed model analysis of physiological parameters during the sAPN and PB.

Parameter	Effect of prone^a^	Effect of QS^a^	Effect of PMA^a^	Position × state^a^	Position × PMA^b^	Chronological age^c^	Position × chronological age^c^
sAPN							
sAPN duration	NS	↑ (0.035)	↓ (< 0.001)	NS	0.034	↓ (0.008)	NS
%TST in sAPN	NS	↓ (< 0.001)	NS	NS	NS	↓ (0.031)	NS
ΔSpO_2_%	↓ (0.002)	↓ (0.001)	↑ (< 0.001)	NS	NS	↑ (< 0.001)	NS
ΔHR%	NS	↓ (0.023)	↑ (0.008)	NS	NS	↑ (0.012)	NS
ΔTOI%	NS	NS	↑ (< 0.001)	NS	NS	↑ (< 0.001)	NS
%TST with SpO_2_ < 90%	NS	NS	↓ (0.004)	< 0.001	< 0.001	↓ (0.018)	0.006
%TST with TOI < 55%	NS	NS	NS	NS	< 0.001	NS	NS
PB							
PB duration	NS	↑ (0.009)	↓ (< 0.001)	NS	NS	↓ (0.003)	NS
%TST in PB	NS	NS	NS	NS	NS	↓ < 0.001	0.031
ΔSpO_2_%	↓ (0.029)	NS	↑ (0.001)	NS	NS	↑ (< 0.001)	0.038
ΔHR%	NS	NS	↓ (< 0.001)	NS	NS	↓ (0.007)	NS
ΔTOI%	NS	NS	↓ (< 0.001)	NS	0.014	↓ (0.040)	0.033
%TST with SpO_2_ < 90%	NS	NS	↓ (0.007)	NS	NS	NS	NS
%TST with TOI < 55%	↓ (0.005)	↓ (0.050)	↓ (< 0.001)	NS	NS	↓ (< 0.001)	< 0.001
Sleep time							
% QS time	↑ (0.017)	N/A	↑ (0.001)	N/A	NS	NS	NS
% AS time	↓ (0.017)	N/A	↓ (0.001)	N/A	NS	NS	NS
FiO_2_							
FiO_2_ > 0.21	↓ (0.005) (AS)	NS	NS	NS	NS	↓ (< 0.001)	(< 0.001)

*Note*: Mixed model analysis of the duration, the percentage change from baseline in HR (ΔHR%); cerebral TOI (ΔTOI%) and SpO (ΔSpO_2_%) during the sAPN or PB episodes; and %TST and FiO_2_. Values represent significant *p*‐values from the three mixed model analyses performed.

Abbreviations: AS, active sleep; FiO_2_, fraction of inspired oxygen; HR, heart rate; PB, periodic breathing; PMA, postmenstrual age; QS, quiet sleep; sAPN, short apneas; SpO_2_, arterial oxygen saturation; TOI, tissue oxygenation index; TST, total sleep time.

^a^ Model 1: position, sleep state, PMA, and position × state interaction.

^b^ Model 2: position × PMA interaction; model included position, sleep state, PMA.

^c^ Model 3: chronological age (weeks 1–10) substituted for PMA; model included position, sleep state, chronological age, and position × chronological age interaction.

### 3.1 Effects of sleep position

#### 3.1.1 Short apneas

The majority of infants experienced sAPN in both sleep positions (Table 2). ∆SpO_2_% was reduced in the prone compared with supine position (main effect, *p* < 0.01), while all other parameters were not affected by the sleep position (Figure 1a–c; Table 4).

#### 3.1.2 Periodic breathing

Overall, fewer infants experienced PB in the prone position, which reached statistical significance at 29–30 weeks and 33–34 weeks PMA during AS (Table 2). In the prone position, the fall in SpO_2_% was reduced (main effect, *p* < 0.05), with post hoc significance at week 5 of age (position × chronological age, *p* < 0.05), and the time spent with TOI < 55% was less compared with supine (main effect, *p* < 0.01; Figures 1d and 2f; Table 4).

For both sAPN and PB, sleep position did not affect the %TST spent in these respiratory events (Figure 2a,d) or the event durations (Table 4). The proportions of infants who experienced ∆SpO_2_ < 90% or ∆TOI < 55% due to sAPN or PB are shown in Table 5. The prone position had little impact on these proportions, and only reduced the proportion of infants with low SpO_2_ during AS at 35–36 weeks PMA for sAPN, and at 33–34 weeks PMA for PB.

**TABLE 5:** The incidence of systemic (SpO_2_ < 90%) or cerebral (TOI < 55%) hypoxia during sAPN and PB in each sleep position and state.

Postmenstrual age (weeks)		Infants that spent time with SpO_2_ <90%				Infants that spent time with TOI <55%			
		AS‐S	QS‐S	AS‐P	QS‐P	AS‐S	QS‐S	AS‐P	QS‐P
25–26 (*N* = 3)	sAPN	3 (100%)	3 (100%)	2 (67%)	2 (67%)	1 (33%)	1 (33%)	1 (33%)	1 (33%)
	PB	3 (100%)	2 (100%)	2 (100%)	1 (100%)	1 (33%)	1 (50%)	1 (50%)	1 (100%)
27–28 (*N* = 11)	sAPN	10 (91%)	8 (73%)	7 (64%)	5 (50%)	3 (27%)	3 (27%)	2 (18%)	0 (0%)
	PB	10 (100%)	4 (57%) ^#^	7 (70%)	3 (60%)	4 (40%)	0 (0%)	3 (30%)	2 (40%)
29‐30 (*N* = 20)	sAPN	12 (60%)	6 (32%)	8 (42%)	8 (42%)	4 (20%)	3 (16%)	3 (16%)	0 (0%)
	PB	15 (79%)	6 (55%)	7 (50%)	6 (60%)	3 (16%)	1 (9%)	4 (29%)	1 (10%)
31–32 (*N* = 29)	sAPN	25 (86%)	13 (50%) ^##^	23 (79%)	8 (30%) ^###^	11 (38%)	7 (27%)	8 (28%)	5 (19%)
	PB	20 (80%)	13 (68%)	12 (57%)	9 (90%)	7 (28%)	6 (32%)	5 (24%)	1 (10%)
33–34 (*N* = 27)	sAPN	24 (89%)	13 (52%) ^##^	22 (81%)	10 (40%) ^##^	10 (37%)	4 (16%)	7 (26%)	3 (12%)
	PB	22 (92%)	13 (72%)	10 (63%) *	8 (73%)	6 (25%)	6 (33%)	4 (25%)	2 (18%)
35–36 (*N* = 19)	sAPN	16 (84%)	8 (42%) ^##^	10 (53%) *	9 (50%)	7 (37%)	5 (26%)	4 (21%)	3 (17%)
	PB	11 (79%)	8 (80%)	8 (67%)	8 (73%)	5 (36%)	3 (30%)	2 (16%)	1 (9%)

*Note*: * = position, *p < 0.05; ^#^ = sleep state, ^#^ *p* < 0.05, ^##^ *p* < 0.01, ^###^ *p* < 0.001.

Abbreviations: AS‐S, active sleep in supine position; AS‐P, active sleep in prone position; PB, periodic breathing; PMA, postmenstrual age; QS‐P, quiet sleep in prone position; QS‐S, quiet sleep in supine position; sAPN, short apneas; SpO_2_, arterial oxygen saturation; TOI, tissue oxygenation index.

**FIGURE 3:**
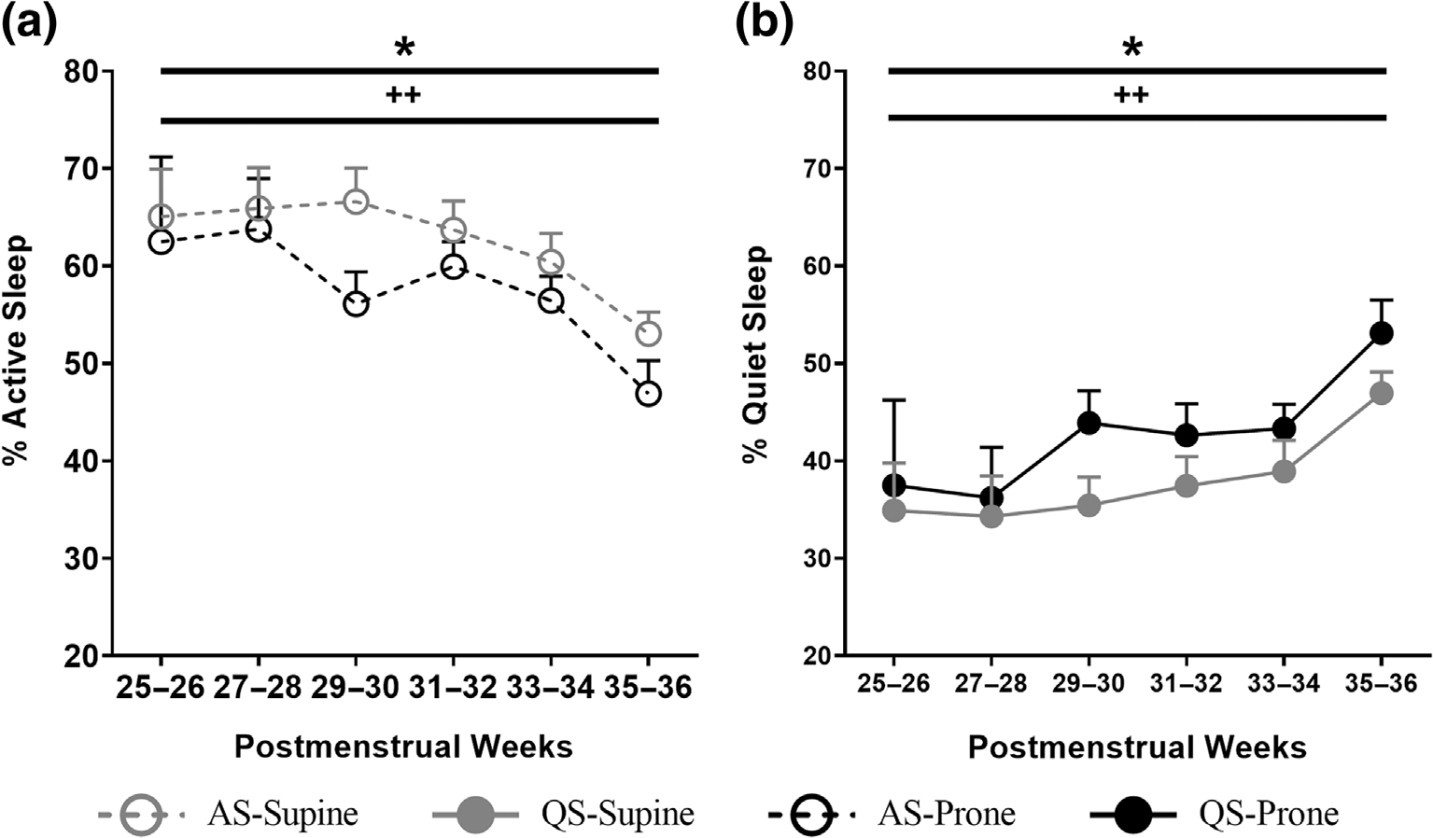
Effect of sleep position and PMA on percentage of time spent in AS (a) and QS (b). Values are mean ± SEM. *p < 0.05 overall effect of sleep position; ^++^ *p* < 0.01 overall effect of PMA. AS, active sleep; PMA, postmenstrual age; QS, quiet sleep.

The FiO_2_ requirement was lower in prone compared with supine (main effect, *p* < 0.01), and post hoc analysis identified significance at weeks 3 and 4 of age (position × chronological age, *p* < 0.001; Table 4). The %QS increased and %AS decreased in the prone compared with supine position (main effect, *p* < 0.05; Figure 3; Table 4).

For the parameters that showed significant changes with the sleep position, none had any significant interactions between position and state, or between position and PMA.

### 3.2 Effects of sleep state

#### 3.2.1 Short apneas

The majority of infants experienced sAPN in both sleep states (Table 2). During QS, the △SpO_2_%, △HR% (Figure 1a,b) and %TST spent in sAPN (Figure 2a) were decreased compared with AS (main effect, *p* < 0.05; Table 4), despite a longer duration of sAPN in QS (main effect, *p* < 0.05, Table 4). Overall fewer infants spent time with SpO_2_ < 90% in QS compared with AS (*p* < 0.05 at 31–32 weeks and 33–34 weeks PMAs in both supine and prone positions, and 35–36 weeks PMA in supine; Table 5).

#### 3.2.2 Periodic breathing

Overall, fewer infants experienced PB during QS compared with AS (*p* < 0.05 at 27–28 and 31–32 weeks PMA in prone position; and 29–30 and 33–34 weeks PMA in supine position; Table 2). The %TST spent in PB was not affected by sleep state; however, the duration of PB episodes was longer in QS than in AS (main effect, *p* < 0.01; Table 4). During QS, the %TST with TOI < 55% was reduced compared with AS (main effect, *p* < 0.05; Figure 2f; Table 4).

### 3.3 Effects of age

#### 3.3.1 Short apneas

The majority of infants experienced sAPN across all PMAs (Table 2). The fall in SpO_2_, HR and TOI increased with increasing PMA and chronological age (main effect, *p* < 0.01; Figure 1a–c; Table 4). However, the time spent with SpO_2_ < 90% (Figure 2b) and the duration of sAPN (Table 4) decreased with increasing PMA and chronological age (main effect, *p* < 0.05).

#### 3.3.2 Periodic breathing

There was a trend of fewer infants experiencing PB with increasing PMA (Table 2; *p* = 0.07 for AS in supine position). The △SpO_2_% increased, but the △HR% and △TOI% decreased with increasing PMA and chronological age (main effect, *p* < 0.01; Figure 1d–f; Table 4). The time spent with SpO_2_ < 90% or TOI < 55% (Figure 2e,f), and the duration of PB (Table 4) also decreased with increasing PMA and chronological age (main effect, *p* < 0.01).

The %QS increased and %AS decreased with increasing PMA (main effect, *p* < 0.01; Figure 3; Table 4).

## 4 DISCUSSION

To our knowledge, this is the first longitudinal study to investigate the effects of sleep position, sleep state and PMA on sAPN and PB, and the associated physiological changes, including cerebral oxygenation in hospitalized preterm infants. We found that the prone position only reduced the severity of desaturations for sAPN. The prone position had more effects on PB, reducing the proportion of infants that exhibited PB, severity of desaturations and time spent in cerebral hypoxia. Sleep state, rather than position, had more marked effects on sAPN. During QS, less time was spent in sAPN, falls in HR and desaturations were less severe, and the proportion of infants with sAPN‐induced systemic hypoxia was reduced compared with AS. QS was also associated with fewer infants having PB and reduced time spent in PB‐induced cerebral hypoxia. Notably, the percentage of QS was higher in the prone compared with supine position, irrespective of PMA. With increasing PMA, respiratory event duration and time spent in systemic hypoxia for sAPN decreased, but falls in SpO_2_, HR and TOI increased. In contrast to our findings during sAPN, PMA generally improved respiratory stability during PB, except desaturations, which worsened with age.

In the NICU, preterm infants are often slept prone as it has been associated with improved respiratory stability (Barsman et al., 2015). However, we did not identify any position‐related differences in sAPN or PB event duration, time spent in sAPN/PB or falls in HR and TOI. Prone positioning decreased the severity of desaturations in both sAPN and PB, and reduced time spent in cerebral hypoxia during PB. Supplemental oxygen, as adjusted by NICU staff to maintain SpO_2_ levels within the clinical target range, was significantly higher in the supine versus prone position. This may have masked the severity of desaturations in the supine position, thereby reducing some of the desaturation differences between the prone and supine positions. The less severe desaturation in the prone position could be attributable to gravity‐related mechanisms, such as reduced chest wall distortion (Leipala et al., 2003) and thoraco‐abdominal asynchrony (Gouna et al., 2013), which increase tidal volume and improve the ventilation/perfusion ratio (Wagaman et al., 1979). Pulmonary resistance is lower in the prone position due to improved rib cage stabilization, rendering the preterm airway less susceptible to collapse and compression (Bhat et al., 2006; Mendoza et al., 1991). Notably, prone sleeping may be associated with negative effects in preterm infants. We have previously reported increased cerebral fractional oxygen extraction in extremely preterm infants in the prone position in the first week after birth (Shepherd et al., 2019), suggestive of low cerebral blood flow during this high‐risk period of intraventricular haemorrhage. The prone position has also been associated with low cardiac output (Ma et al., 2015), and compression of vertebral arteries and ipsilateral jugular vein (Pamphlett et al., 1999) due to the lateral head and neck rotation.

Other studies examining preterm infants prior to (Mendoza et al., 1991; Shepherd et al., 2020) and following hospital discharge (Bhat et al., 2006) have reported reduced severity of bradycardias and desaturations together with reduced apnea frequency in the prone position. However, in studies where sleep state was controlled for, there was an absence of position‐related differences (Elder et al., 2005; Elder et al., 2011). Our analyses accounted for the effects of sleep state and found that QS, rather than the prone position, improved the physiological parameters related to sAPN and PB. QS reduced time spent in sAPN with less severe falls in SpO_2_ and HR, and a reduced proportion of infants having PB with less time spent in cerebral hypoxia, compared with AS. Importantly, QS is increased and AS is decreased in prone sleeping. QS is the state of improved cardiorespiratory stability (Bhat et al., 2006; Elder et al., 2005), while AS is associated with reduced skeletal muscle tone (Aserinsky & Kleitman, 1953), less coordinated diaphragmatic contraction (Gaultier, 1995), increased chest wall asynchrony (Stokes et al., 1989), irregular respiration (Caruana‐Montaldo et al., 2000) and more variable and lower SpO_2_ (Gaultier & Gallego, 2005) in preterm infants. Similarly, TOI is lower in AS than QS in preterm infants (Fyfe et al., 2014; Shepherd et al., 2019), due to increased cerebral oxygen consumption and the endogenous brain activation characteristic of AS (Tarullo et al., 2011). Accordingly, the bedside observations and reported benefits associated with the prone position may be attributed to the increased time spent in QS rather than the position itself. Notably, AS is critical for brain maturation, through the formation and pruning of synapses and memory consolidation (Tarullo et al., 2011). The importance of AS is exemplified by changes in sleep state organization as preterm infants typically spend 80% of TST in AS at 29–30 weeks GA, which decreases to 55% by term‐equivalent age (Barbeau & Weiss, 2017). Moreover, animal studies have identified that AS/rapid eye movement sleep deprivation during the neonatal period is correlated with altered neurotransmitter sensitivity, reduced cerebral cortex and brainstem volume (Mirmiran et al., 1983) and cognitive complexity (Lesku et al., 2006). Neonates with reduced AS show impaired developmental and cognitive outcomes at 6 months of age (Arditi‐Babchuk et al., 2009). Hence, reducing AS by placing preterm infants prone (for cardiorespiratory stability) could potentially have adverse implications on brain maturation.

In contrast to our hypotheses, increasing PMA, in general, had negative effects on sAPN parameters. As this study was conducted in a tertiary centre, a likely reason for this observation is the early transfer of clinically stable preterm infants to regional special care facilities closer to home. As such, those included at later PMAs were more likely to be infants with persistent cardiorespiratory instability. Nevertheless, an increasing proportion of infants at the later PMAs were taken off ventilatory support (51% at 31–32 weeks, 70% at 33–34 weeks, and 78% at 35–36 weeks PMA), coinciding with the worsening of physiological parameters related to sAPN and PB. Removal of ventilatory support could result in de‐recruitment of lung volume, partial atelectasis and ventilation‐perfusion mismatch, which may contribute to the more severe physiological impact from sAPN and PB at later PMAs (van Delft et al., 2020). Another study by our group in a different cohort of preterm infants also found that preterm infants who had less time on ventilatory support before 32 weeks PMA spent more time in PB at the late preterm age, and were at risk for low cerebral oxygenation during PB (Yee, Siriwardhana, Nixon, et al., 2023). These findings indicate that sAPN and PB are often not considered in the clinical decisions to discontinue ventilatory support. Intermittent hypoxia, as observed during the sAPN and PB, is known to cause systemic and brain inflammation in rat pups (Darnall et al., 2017) and poor cognitive outcomes in human studies (Poets et al., 2015). Even mild intermittent hypoxia can cause permanent neurofunctional deficits and hypomyelination (Juliano et al., 2015). Although the averaged falls in physiological parameters in our study were small, several infants had falls in HR and SpO_2_ of >20% and TOI of >10% from baseline during sAPN. These falls were even more severe during PB, reaching >45% for HR and SpO_2_, and >20% for TOI. Similar findings were observed for the %TST in systemic and cerebral hypoxia. During sAPN and PB, respectively, individual infants spent up to 14% and 35% TST with systemic hypoxia (SpO_2_ < 90%); and up to 30% and 53% TST with cerebral hypoxia (TOI < 55%). A 10% reduction in cerebral oxygenation in preterm infants is of clinical concern, and time spent with low cerebral oxygenation of < 55% has been associated with adverse neurodevelopmental outcomes (Alderliesten et al., 2013; van Bel et al., 2008). In addition, increased time spent in sAPN and PB correlates with reduced language and motor scores at 6 months correlated age (Yee, Siriwardhana, Nixson, et al., 2023). These respiratory events and the associated intermittent hypoxia are often treated with prone sleeping (Barsman et al., 2015), which, as we have shown, effectively improves the cardiorespiratory stability by increasing QS and decreasing AS. Given the key role of AS in brain maturation, our findings raise an important question of whether these respiratory events should be managed with ventilatory support rather than prone sleeping.

Interestingly, we observed that sAPN and PB were influenced differently by the sleep position, state and PMA, even though PB is often viewed as repetitive sAPN. PB reflects immature/impaired chemoreceptor reactivity and “high loop gain” with overcompensation to oscillating pO_2_ and pCO_2_ levels (Khoo et al., 1982). PB is typically limited to infancy and resolves with age for both preterm and term born infants, while apnea is a common manifestation of various respiratory dysfunctions across many population groups. The differences we observed indicate that PB is physiologically distinct from sAPN.

The major strength of our study was the longitudinal design with all infants studied for ≥ 6 weeks after birth, in both sleep positions and both sleep states. Limitations of our study were the small sample size with inclusion of only clinically stable preterm infants as evidenced by their oxygen requirement of < 30%. The infants studied at later PMAs and chronological ages did not include those transferred to regional nurseries. We could not distinguish between central apneas, mixed apneas and obstructive apneas in this study due to the lack of nasal airflow measurement, which was not possible as infants often already had nasal continuous positive airway pressure (CPAP) or high flow for ventilatory support as well as nasogastric feeding tubes. In addition, PB events are less common when compared with sAPN, therefore for certain sleep state–position combinations only a few infants were included for the PB analyses. The impact of different ventilatory support was not addressed in this study due to inadequate power. Further studies with larger populations and including infants receiving higher level of respiratory support or oxygen are warranted to confirm the findings and clinical recommendations proposed in this study.

In conclusion, our study highlights that when sleep state is controlled for, the prone sleeping position has relatively little benefit in improving the frequency and physiological consequences of both sAPN and PB. QS improves cardiorespiratory stability and is increased in the prone position at the expense of AS, which is critical for brain maturation. Whilst cardiorespiratory instability appeared minimal amongst averaged group results, individual infants exhibited marked falls in physiological parameters during sAPN and PB despite being deemed clinically stable. These findings add to the growing body of research suggesting that these brief respiratory pauses may not be benign, and that prone positioning may not be an appropriate management strategy in preterm infants.

## AUTHOR CONTRIBUTIONS

**Georgina Plunkett:** Writing – original draft; methodology; writing – review and editing; formal analysis. **Stephanie Yiallourou:** Conceptualization; methodology; writing – review and editing; data curation; funding acquisition. **Aimee Voigt:** Formal analysis; methodology; writing – review and editing. **Aishah Segumohamed:** Formal analysis; writing – review and editing. **Kelsee Shepherd:** Investigation; methodology; formal analysis; writing – review and editing. **Rosemary Horne:** Conceptualization; investigation; methodology; writing – review and editing; supervision; funding acquisition. **Flora Wong:** Conceptualization; investigation; funding acquisition; writing – original draft; methodology; validation; writing – review and editing; project administration; data curation; supervision; resources.

## FUNDING INFORMATION

This work was supported by project grant funding from the National Health and Medical Research Council (NHMRC) of Australia (Project No. 1083026), and the Victorian Government's Operational Infrastructure Support Program; F.Y.W. was supported by NHMRC MRFF Career Development Fellowships 1159120 and the Victor Yu Clinical Research Fellowship.

## CONFLICT OF INTEREST STATEMENT

The authors declare no conflicts of interest.

## PATIENT CONSENT

Written informed parental consent was obtained.

## Data Availability

The data that support the findings of this study are available on request from the corresponding author. The data are not publicly available due to privacy or ethical restrictions.
